# Methylphenidate and sustained attention in rats: a systematic review

**DOI:** 10.1007/s00213-026-07007-w

**Published:** 2026-02-13

**Authors:** Adela Batanero-Geraldo, Fátima Montiel-Herrera, Juan Carlos López, Juan Pedro Vargas

**Affiliations:** Departamento de Psicología Experimental, Facultad de Psicología, Universidad de Sevilla. C/Camilo José Cela, S/N, 41018 Seville, Spain

**Keywords:** Methylphenidate, Sustained attention, Behavioral tests, Rats, Cognitive processes

## Abstract

**Rationale:**

Methylphenidate (MPH) is widely used to study attentional processes in animal models, particularly in rats, due to the controlled experimental conditions and the ability to assess its neurobiological effects. Behavioral tasks analogous to those used in human studies provide valuable insights into the drug’s impact on attention. While many studies have investigated its acute effects or its long-term influence on neurotoxicity and sensitization, no comprehensive review has synthesized these findings within a unified framework.

**Objectives:**

This systematic review aims to analyze the available evidence on the effects of MPH on attention in rats, considering the behavioral tests used and the administered doses.

**Methods:**

A systematic literature search identified studies published between 2014 and 2024, selecting 20 relevant articles that assessed attention and related processes through various behavioral paradigms. This review was pre-registered in PROSPERO (CRD42024563124).

**Results:**

The findings reveal that low doses of MPH enhance attentional performance and working memory, whereas high doses impair attention and increase locomotor activity. Additional factors, such as stress, age at administration, and route of administration, significantly influence the drug’s effects. In particular, Wistar and Wistar-Kyoto rat strains appear to be the most susceptible to its influence.

**Conclusion:**

These results highlight the complexity of MPH’s impact on attention and related cognitive processes. The variability in outcomes underscores the need for further research to clarify dose–response relationships and the role of individual and environmental factors in shaping the drug’s effects.

**Supplementary Information:**

The online version contains supplementary material available at https://doi.org/10.1007/s00213-026-07007-w.

## Introduction

Attention is a fundamental cognitive process crucial for adaptation and proper functioning, enabling the selection and processing of relevant information based on environmental demands (Xia et al. 2024). In preclinical research, attention is typically defined as the set of neurocognitive operations that regulate the selective allocation of processing resources toward task-relevant stimuli while suppressing competing inputs (Posner & Petersen 1990; Sarter et al. 2001). Deficits in its normal activity are a feature of various psychiatric and neurological disorders, such as schizophrenia, depression, addiction, and attention deficit hyperactivity disorder (ADHD) (Quintero et al. 2022).

Within attentional mechanisms, a common distinction is made between voluntary and involuntary processes, which modulate stimulus capture according to perceived relevance. Top-down attention reflects voluntary processes guided by task representations, which set a bias for selecting the goal-relevant stimulus. In contrast, bottom-up attention reflects involuntary processes in which a salient stimulus captures the mind, creating a different bias driven by its physical prominence (Anderson 2021b; Wu 2024).

One such mechanism is value-driven attention, which facilitates the prioritization of reward-associated stimuli. This form of goal-directed attentional control is influenced by outcomes, leading individuals to prioritize high-reward-value stimuli. This reward-predictive learning mechanism is highly adaptive, as it provides a prospective understanding of the relationship between stimuli (Anderson 2021a; Huang et al. 2023). However, in some cases, this mechanism could lead to maladaptive patterns affecting cognitive and behavioral performance (Salmon et al. 2024; Wu 2024). That is, previously reward-associated stimuli may continue to capture attention even when they are no longer relevant to the ongoing task. This can result in excessive or insufficient attentional bias to previously reward-associated stimuli, even when they are no longer task-relevant, disrupting information processing. For example, in depression, individuals tend to show reduced value-driven attentional capture, suggesting lower sensitivity to previously reinforced stimuli. In contrast, studies in children with ADHD have shown that they update their attentional priorities faster than controls when previously rewarded stimuli are no longer associated with reward (Anderson 2021b).

The attentional deficits observed in these disorders are closely linked to alterations in executive functions and underlying neurochemical systems. Attention is supported by interactions between frontoparietal and frontostriatal networks, with the medial prefrontal cortex (mPFC) playing a central role in cue detection, top-down control, and maintenance of attentional effort (Dalley et al. 2004). Neurotransmitters such as dopamine (DA), norepinephrine (NE), serotonin (5-HT), glutamate, and gamma-aminobutyric acid (GABA) play a key role in attentional modulation (Quintero et al. 2022). Cholinergic projections from the basal forebrain modulate vigilance and signal amplification (Sarter et al. 2005), whereas catecholaminergic inputs, especially dopamine and norepinephrine, optimize prefrontal cortical function by enhancing signal-to-noise ratios (Arnsten 2009). Specifically, DA is crucial for regulating motivation and stimulus value assignments. A deficit in dopaminergic transmission can lead to reduced motivation and, consequently, attentional difficulties associated with disorders such as ADHD and depression (Quintero et al. 2022).

ADHD is a neurodevelopmental disorder characterized by deficits in attention, hyperactivity, and impulsivity, which are linked to alterations in neurotransmitters, including DA, NE, 5-HT, glutamate and GABA. In particular, the dopamine transporter (DAT-1) and the norepinephrine transporter (NET) play a crucial role in regulating this disorder (Groom & Cortese 2022). Dysregulation of DA releases and NE reuptake in the extracellular space disrupt higher cognitive processes, such as executive functions (Groom & Cortese 2022; Quintero et al. 2022). One of the main pharmacological treatments for ADHD is methylphenidate (MPH), a central nervous system stimulant that a blocks DAT and NET, increasing dopamine and norepinephrine signaling across fronto-striatal and related cortical circuits, including the prefrontal cortex (PFC). These neuromodulatory systems are sensitive to pharmacological manipulation, making them primary targets through which methylphenidate (MPH) exerts its effects on attentional performance (Arnsten 2009) MPH has been shown to reduce ADHD symptoms and improve attentional control and behavioral regulation (Groom & Cortese 2022; Quintero et al. 2022).

However, long-term administration is often associated with several side effects, including neurotoxicity, alterations in neuronal plasticity, and the development of tolerance and dependence (Cherkasova & Hechtman 2009). Additionally, MPH effects may vary depending on dose and treatment duration, leading to discrepancies in experimental findings. At the neurobiological level, chronic MPH use can induce changes in key DA circuits, including the mesolimbic and mesocortical systems, affecting regions such as the ventral tegmental area, frontal lobe, amygdala, and hippocampus (Goto & Grace 2008; Zetterström et al. 2022).

The study of MPH effects on attention has been extensively addressed using animal models, particularly in rats, due to the possibility of controlling experimental variables and accurately assessing their neurobiological impact (Salmon et al. 2024). To assess attention in rodents, behavioral tests analogous to those used in humans have been employed, such as the 5-Choice Serial Reaction Time Task (5-CSRTT), the Sustained Attention Task (SAT), the 5-Choice CPT (5C-CPT), and the rodent CPT (rCPT). Human research commonly relies on Continuous Performance Tests (CPTs) such as the Conners’ CPT, the TOVA, or the X-CPT, which evaluate sustained attention and response control (Groom & Cortese 2022). Each rodent task allows for the evaluation of different aspects of attention, although they show methodological differences. For example, the 5-CSRTT is the most widely used but lacks signal-free trials, hindering the application of signal detection theory (SDT) used in human studies. SAT, on the other hand, includes these trials, allowing for more accurate measurement of attentional performance. The 5C-CPT evaluates both attention and impulsivity, making it more consistent with human tests. Importantly, the rCPT introduces touchscreen-based symbolic stimuli, closer to those used in human CPTs, which improves cross-species comparability (Groom & Cortese 2022; Salmon et al. 2024).

While there is extensive literature on MPH effects on animal behavior, available studies tend to focus on its acute impact or chronic effects on neurotoxicity and sensitization in isolation. A comprehensive analysis synthesizing these findings within the same experimental framework has not been conducted yet. Therefore, this systematic review is aimed at analyzing the available evidence on MPH effects on rat attention, considering the behavioral tests employed and administered doses. The results obtained could have significant implications for understanding MPH's impact on attentional processes. This knowledge may aid in refining pharmacological treatments for individuals with ADHD and other disorders, enabling a more precise evaluation of its benefits and risks within a therapeutic context.

The effects of MPH vary depending on the cognitive process studied. Although this systematic review focused on attentional processes, articles evaluating executive functions with references to attentional processes were included. This is because executive functions, such as working memory and behavioral flexibility, interact dynamically with attention and contribute to overall cognitive control. Therefore, this review encompasses findings not only on sustained attention but also on related executive domains that influence or are influenced by attentional mechanisms.

## Method

This study was conducted in accordance with the 2020 Preferred Reporting Items for Systematic Reviews and Meta-Analyses (PRISMA) guidelines (Page et al., 2021). The study protocol was prospectively registered in the International Prospective Register of Systematic Reviews (PROSPERO: CRD42024563124) to ensure methodological transparency and minimize bias.

To manage and organize the retrieved literature, we used Zotero, a reference management software, which facilitated systematic storage, citation tracking, and removal of duplicate records.

### Bibliographic search

The literature search was conducted between January 31, 2024, and March 5, 2024, across four major databases: PsycInfo, Web of Science (WOS), SCOPUS, and PubMed. The search strategy included the keywords *methylphenidate*, *MPH*, *animal models*, *rats*, *psychology*, *experimental study*, and *sustained attention*. A two-phase search strategy was implemented to optimize the retrieval of relevant studies. The first phase involved broad keyword combinations, including (methylphenidate and animal models, methylphenidate and experimental study, methylphenidate and rats). In addition to methylphenidate we use the acronym MPH, returning the same result. This initial search yielded 33,661 articles across the four databases.

To refine the results and focus on the primary outcome of interest, a second search was conducted, incorporating the keyword *sustained attention* into the queries (“sustained attention” and methylphenidate and rats) and (“sustained attention" and (methylphenidate and rats)), identifying an additional 95 articles. This brought the total number of retrieved records to 33,756 (PubMed: *n* = 1,370; PsycInfo: *n* = 26,345; WOS: *n* = 1,842; SCOPUS: *n* = 4,199).

### Inclusion and exclusion criteria

Inclusion and exclusion criteria were then applied to ensure alignment with the study’s primary objectives. We included only experimental studies conducted in rats, published in peer-reviewed scientific journals between 2014 and 2024. This temporal range was chosen based on a PubMed search showing a marked increase in publications on methylphenidate and attention in animal models from 2014 onward. The studies had to be written in any language, assess attentional or related tasks with MPH administration, and be accessible in full text. (see Table 1).

**Table 1:** Inclusion and exclusion criteria

Criterion (Order of Application)	Reason	Inclusion criteria	Exclusion criteria
1	Document Type	Peer-reviewed scientific articles published in journals	Books, book chapters, theses
2	Methodology	Experimental studies in animal models	Studies in humans, non-experimental studies
3	Publication Year	2014–2024	Pre-2013
4	Access	Accessible articles	Inaccessible articles
5	Population	Rats	Other animals or humans
6	Phenomenon of Interest	Attentional tasks with MPH administration	Other phenomena

Inclusion and exclusion criteria were based on the review’s objectives. Studies not meeting these criteria were excluded

**Fig. 1:**
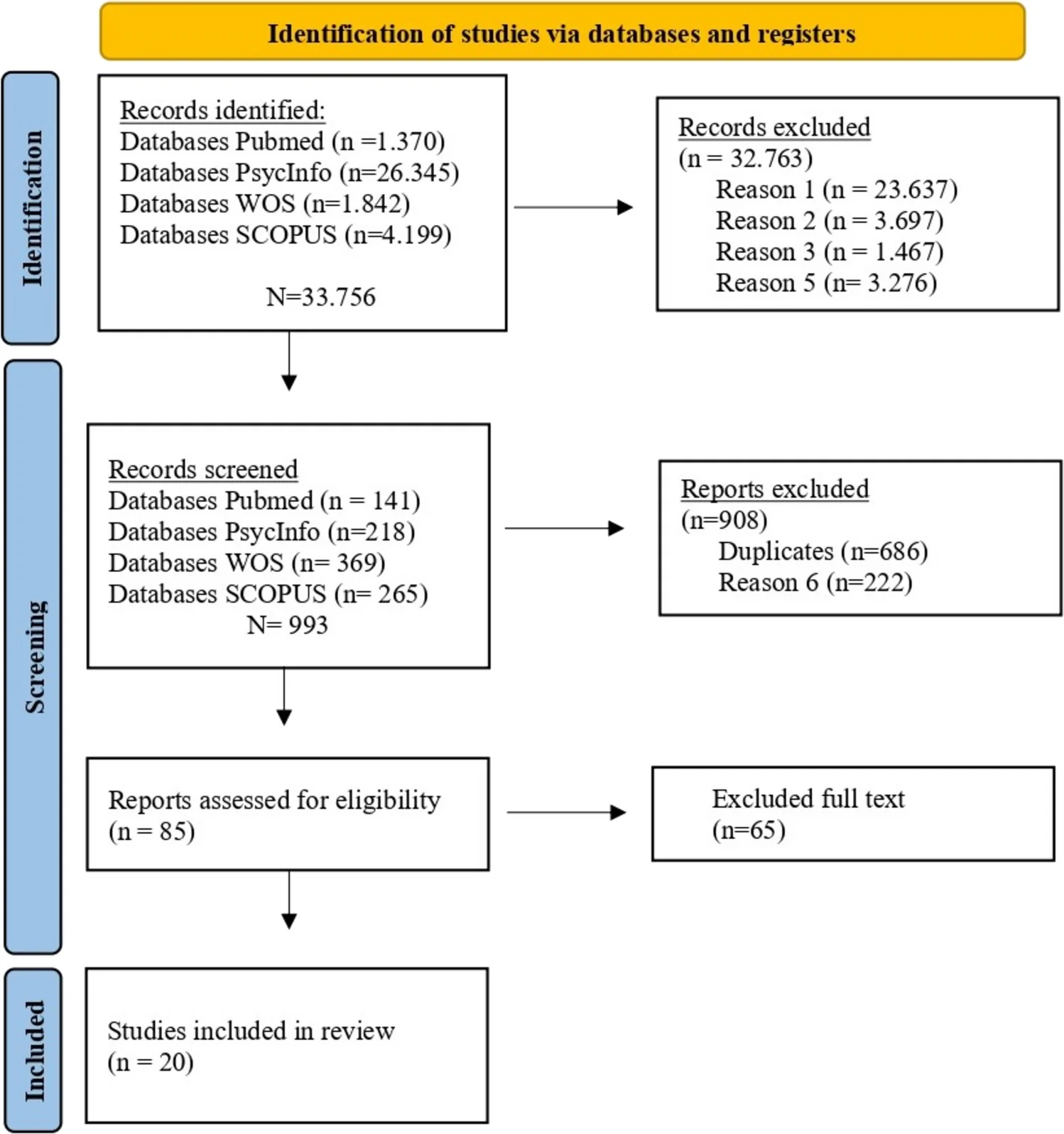
PRISMA 2020 flow diagram. *Note. *From Page et al. (2021). Distributed under the terms of the Creative Commons Attribution License (CC BY 4.0)

Two independent reviewers conducted the study selection process in three stages: removal of duplicates, title and abstract screening, and full-text review. Disagreements were resolved through consensus. A third reviewer was available to adjudicate unresolved discrepancies; however, no disagreement required escalation to this third reviewer. The application of inclusion and exclusion criteria, along with abstract screening, reduced the selection to 87 articles. These articles were subjected to a detailed full-text review, during which studies not using MPH, behavioral tests related to attention, or attentional measures were excluded. Articles using other rodents were also discarded. Initially, studies using only male rats were planned for exclusion, but this criterion was abandoned due to the scarcity of articles meeting this requirement. Ultimately, 20 articles were included in the final sample (Fig. 1).

### Data extraction

The data extraction from the selected articles was performed manually. A table (Appendix C) was created to summarize the items considered most relevant by the reviewers, selected to allow the simplest possible comparison across studies given their heterogeneity, including subjects, behavioral tests, drugs, and dosages. Given the variability of the studies, only the most representative data influencing the results and conclusions were extracted.

To assess study quality, the Systematic Review Center for Laboratory Animal Experimentation (SYRCLE) risk of bias tool was used. Two reviewers independently evaluated each study, and discrepancies were discussed until consensus was reached. Ten items related to selection bias, performance bias, attrition bias, reporting bias, and other biases were assessed. Each study was classified as having low, uncertain, or high risk of bias for each item (Appendix A). Additionally, the Collaborative Approach to Meta-Analysis and Review of Animal Data from Experimental Studies (CAMARADES) checklist was used to evaluate experimental design quality, including randomization, blinding, sample size, and detailed reporting of methods and results (Appendix B).

A meta-analysis was not conducted due to high heterogeneity among studies. Only nine studies specifically assessed attention in animal models (Andrzejewski et al. 2014; Beaudin et al. 2024; Bhattacharya et al. 2015; Chu et al. 2016; Ding et al. 2018; Higgins et al. 2020; Spencer & Berridge 2019; Takahashi et al. 2018; Tomlinson et al. 2014), with substantial differences in animal age, task duration, drug administration, and dosing. These inconsistencies limited statistical comparison, despite some overlap in measures and tasks. Consequently, a detailed evidence synthesis was provided in a summary table following the TIDieR template, presented in Appendix C (Hoffmann et al. 2017).

To provide a clearer overview of the studies considered for a potential meta-analysis, we have included a summary table (Appendix D). This table highlights the nine studies that specifically assessed attention, indicating the statistical significance of their findings and the potential risk of bias due to sample size.

## Results

A total of 20 peer-reviewed studies published in English between 2014 and 2024 were included in this systematic review, examining the effects of methylphenidate (MPH) on attention in experimental rat models. Figure 1 shows a PRISMA flow diagram detailing the study selection process, from initial search to final inclusion. Key data from each study, including sample size, behavioral tests, drug administration methods, dosages, and other relevant variables, are summarized in a structured table (Appendix C). The findings were analyzed narratively, providing a comprehensive synthesis of the results in the context of the research objectives.

### Drug administration and dosage

The reviewed studies employed diverse methods of MPH administration, including bilateral brain cannulas (Spencer & Berridge 2019), oral gavage (Alfadly et al. 2014; Zhou et al. 2017), subcutaneous injections (Comeau & Kolb 2020; Takahashi et al. 2018), intraperitoneal injections (Andrzejewski et al. 2014; Ding et al. 2018; Galizio et al. 2014; Higgins et al. 2020; Motamedi et al. 2019; Ravichandran et al. 2015; Rowan et al. 2015; Tomlinson et al. 2014), oral administration via pipette (Haleem et al. 2015; Kim et al. 2016; Zubedat et al. 2015), and oral delivery through cookies (Beaudin et al. 2024; Bhattacharya et al. 2015) or strawberry shakes (Benn & Robinson 2024). Most studies administered MPH 30 min before behavioral testing (Alfadly et al. 2014; Andrzejewski et al. 2014; Beaudin et al. 2024; Benn & Robinson 2024; Ding et al. 2018; Higgins et al. 2020; Motamedi et al. 2019; Ravichandran et al. 2015; Takahashi et al. 2018; Tomlinson et al. 2014), while others used a 15-min pre-test interval (Bhattacharya et al. 2015; Chu et al. 2016; Galizio et al. 2014). Some studies implemented chronic administration protocols, ranging from 25 to 36 days, with behavioral tests conducted after withdrawal periods (Comeau & Kolb 2020; Rowan et al. 2015; Zubedat et al. 2015).

MPH dosages varied widely, from 0.1 mg/kg to 16 mg/kg and were categorized as low (0.1–3 mg/kg), medium (4–8 mg/kg), or high (9–30 mg/kg). Low doses were most prevalent, with 0.5 mg/kg and 1 mg/kg being the most frequently used. Medium and high doses were less common and usually tested in comparison with low doses. Rowan et al. (2015) uniquely employed a single high dose of 20 mg/kg.

### Control groups and comparative substances

Nine studies used saline as a control alongside MPH (Beaudin et al. 2024; Bhattacharya et al. 2015; Chu et al. 2016; Comeau & Kolb 2020; Haleem et al. 2015; Kim et al. 2016; Ravichandran et al. 2015; Rowan et al. 2015; Zubedat et al. 2015), while the remaining 11 studies included additional substances such as atomoxetine and amphetamine for comparative analysis (Alfadly et al. 2014; Andrzejewski et al. 2014; Benn & Robinson 2024; Ding et al. 2018; Galizio et al. 2014; Higgins et al. 2020; Motamedi et al. 2019; Spencer & Berridge 2019; Takahashi et al. 2018; Tomlinson et al. 2014; Zhou et al. 2017).

### Behavioral tasks and cognitive performance

The reviewed studies employed a variety of behavioral tasks to assess the effects of MPH on attention and related cognitive functions in rats. This section begins with a detailed analysis of sustained attention before discussing how other cognitive domains interact with these attentional processes.

#### Sustained attention tasks

To assess sustained attention, various operant tasks were employed across the included studies. These tasks share a common objective of measuring the ability to maintain focus on relevant information over extended periods, though they differ in their specific methodological approaches and the aspects of attention they emphasize.

The SAT, employed in four studies (Andrzejewski et al. 2014; Bhattacharya et al. 2015; Chu et al. 2016; Spencer & Berridge 2019), requires rats to detect target stimuli (signals) presented with a predetermined probability, interspersed with signal-free trials. Most studies using signal detection tasks indicate that MPH enhances sustained attention performance (Andrzejewski et al. 2014; Beaudin et al. 2024; Bhattacharya et al. 2015; Chu et al. 2016; Ding et al. 2018; Higgins et al. 2020; Spencer and Berridge 2019; Takahashi et al. 2018; Tomlinson et al. 2014). Notably, Spencer and Berridge (2019) reported that these improvements in sustained attention were pronounced when MPH was infused specifically into the dorsomedial prefrontal cortex, suggesting anatomical specificity of MPH effects on attention. Additionally, Chu et al. (2016) found cognitive improvements at higher doses in subjects with initially low baseline performance, highlighting the importance of baseline performance level as a moderating variable.

The 5-CSRTT, employed in three studies (Beaudin et al. 2024; Higgins et al. 2020; Tomlinson et al. 2014), presents brief visual stimuli in one of five spatial locations and requires subjects to nose-poke the location matching the stimulus location within a specified time window. The 5-CSRTT differs from SAT in that it lacks signal-absent trials, which limits the application of classic signal detection theory metrics. However, it provides detailed information on the speed–accuracy trade-off and multiple components of attention and inhibitory control.

Overall, the three studies indicate that MPH exerts stronger effects in animals with lower baseline performance. Beaudin et al. (2024) reported improved signal detectability and reduced premature responses at low doses (1 mg/kg), along with an inverted U-shaped dose–response curve for both sustained attention and impulsivity. Tomlinson et al. (2014) found that MPH enhances attentional performance in “Low-Attentive” rats and reduces impulsive responding in “High-Impulsive” rats. Finally, Higgins et al. (2020) concluded that the effects of MPH depend on the animals’ baseline functional profile, reinforcing the idea that its efficacy is modulated by initial attentional and impulsivity levels.

The CPT and its rodent variants (5C-CPT and rCPT) assess both attention and impulsivity by requiring subjects to respond to target stimuli while withholding responses to non-target stimuli. The 5C-CPT provides a more translational approach to human clinical assessment compared to traditional 5-CSRTT, as it better aligns with human attention paradigms. One study employed CPT variants (Ding et al. 2018), reporting enhanced signal detectability at low MPH doses (1 mg/kg), consistent with findings from SAT studies.

Additional attention assessment paradigms included the Five-Choice Pavlovian Attention Test (5 T-PAT, employed in Takahashi et al. 2018) and the Rodent Rapid Serial Visual Presentation task (R-RSVP, employed in Benn and Robinson 2024). The 5 T-PAT measures attention by requiring discrimination among Pavlovian stimuli, while R-RSVP assesses the ability to detect target stimuli embedded within rapid stimulus streams. Takahashi et al. (2018) reported that MPH has a more pronounced effect in subjects with initially low performance levels. In contrast to most findings, Benn and Robinson (2024) found no significant effects of MPH on attentional performance measures.

Overall, most studies reported attentional improvements following MPH administration (Andrzejewski et al. 2014; Beaudin et al. 2024; Bhattacharya et al. 2015; Chu et al. 2016; Higgins et al. 2020; Kim et al. 2016; Spencer and Berridge 2019; Takahashi et al. 2018; Tomlinson et al. 2014; Zubedat et al. 2015), with low doses (0.5–1 mg/kg) generally proving most effective. However, important exceptions exist. Bhattacharya et al. (2015) found that 6 mg/kg and 8 mg/kg doses improved attention in adult rats, and Chu et al. (2016) reported improvements with 8 mg/kg in animals with low baseline performance.

A critical moderating factor across studies was baseline attentional performance. Multiple authors reported that MPH exerted more pronounced effects in subjects with initially poor attentional function (Beaudin et al. 2024; Chu et al. 2016; Higgins et al. 2020; Takahashi et al. 2018; Tomlinson et al. 2014). In contrast, one study found no significant effects of MPH on attentional measures (Benn and Robinson 2024), highlighting the heterogeneity in outcomes.

Although sustained attention is the primary focus of this review, it operates within an integrated system of executive functions, including working memory (maintaining and manipulating information) and behavioral flexibility (adapting responses to changing demands). These processes rely on overlapping prefrontal networks and interact bidirectionally—for instance, maintaining attention requires working memory, and shifting attention depends on flexibility. Consequently, many attentional paradigms also engage these functions. The following sections discuss how MPH affects these related executive processes and their influence on attention.

#### Working memory

Working memory, defined as the ability to maintain and manipulate goal-relevant information over brief delays, is intrinsically linked to sustained attention (Engle and Kane 2004). Three studies assessed spatial working memory in rats: Comeau and Kolb (2020) and Spencer and Berridge (2019) used a T-maze, whereas Alfadly et al. (2014) employed a radial arm maze (RAM).

Results were contradictory, Spencer and Berridge (2019) reported improved working memory performance with low-dose MPH (0.125 µg/g intracerebral administration), consistent with the observation of attentional improvement in the same study. In contrast, Comeau and Kolb (2020) observed increased errors in MPH-treated subjects using a T-maze, suggesting working memory impairment. Alfadly et al. (2014) found no significant differences between MPH and control groups on spatial working memory measures.

These divergent outcomes may reflect differences in administration age, as Comeau and Kolb (2020) specifically noted that MPH administration from postnatal day (PND) 21 to PND 100 impaired working memory, suggesting that the developmental period during which MPH is administered critically influences outcomes.

#### Behavioral flexibility

Behavioral flexibility, the ability to adapt response strategies when environmental contingencies change, depends critically on attention and working memory (Miyake et al. 2000). Two studies assessed this function using distinct paradigms: Comeau and Kolb (2020) employed juvenile play behavior, whereas Chu et al. (2016) used an attentional set-shifting task.

Results indicated that MPH improved flexibility in subjects with poor baseline performance but impaired high-performing individuals. Specifically, Chu et al. (2016) found that low-dose MPH enhanced set-shifting in poor performers while either having no effect or slightly impairing performance in better-performing animals. This pattern parallels the baseline performance effect observed in sustained attention tasks, suggesting a general principle: MPH may normalize performance in underperforming individuals but produce ceiling effects or even impairment in already high-performing subjects.

#### Motor activity and locomotion

Motor activity in rats has been widely assessed using open field tests and locomotor activity chambers. The relationship between attentional function and motor activity is bidirectional: excessive locomotion can interfere with attentional task performance, while reduced activity may reflect low motivation.

Studies indicate dose-dependent effects of MPH on locomotion. Bhattacharya et al. (2015) reported that the highest dose tested (10 mg/kg) increased locomotor activity, consistent with predictions for high-dose psychostimulants. Similarly, Haleem et al. (2015) observed enhanced motor behavior at 0.5 mg/kg in the activity cage and increased activity in the open field at both 0.5 mg/kg and 1 mg/kg. Comeau and Kolb (2020) corroborated these findings across different behavioral paradigms. In contrast, Zhou et al. (2017) reported that MPH treatment reduced motor hyperactivity, including decreased distance traveled and average velocity.

The relationship between locomotion and anxiety further complicates interpretation. In the open field, Zubedat et al. (2015) observed reduced anxiety in MPH-treated rats, although motor learning capacity was decreased and locomotor activity increased. Kim et al. (2016), using the elevated plus maze, reported that MPH could exacerbate anxiety-like behaviors in some cases.

Overall, these findings underscore that MPH exerts dose- and context-dependent effects on locomotor activity, which can interact with attentional performance and vary according to the behavioral paradigm used.

#### Learning and memory

Learning and memory processes, while distinct from sustained attention, depend on intact attentional allocation and are relevant to understanding the broader cognitive effects of MPH. Regarding learning and memory, MPH was found to enhance avoidance learning (Ravichandran et al. 2015) and spatial memory in some studies (Haleem et al. 2015). However, others reported no significant effects (Galizio et al. 2014; Motamedi et al. 2019), and high doses (≥ 10 mg/kg) occasionally impaired performance (Galizio et al. 2014).

The effects of MPH on inhibitory control have been evaluated using Go/NoGo tasks (Higgins et al. 2020) and differential reinforcement of low rates (DRL) schedules (Andrzejewski et al. 2014). While some studies reported enhancements in response inhibition (Higgins et al. 2020), others observed increased impulsivity (Ding et al. 2018). These divergent outcomes likely reflect moderating factors such as the administered dose of MPH and the baseline behavioral profile of the subjects, highlighting the importance of individual differences and experimental context in interpreting MPH’s effects on inhibitory control.

In summary, the impact of MPH on cognitive abilities in rats varies considerably depending on the behavioral task employed. The most robust and consistent finding concerns sustained attention, where low doses generally enhance performance. However, this enhancement is moderated by baseline performance level and is more pronounced in lower-performing subjects (Beaudin et al. 2024; Chu et al. 2016; Higgins et al. 2020; Takahashi et al. 2018; Tomlinson et al. 2014). Some debate remains regarding the optimal dosage: Andrzejewski et al. (2014), Beaudin et al. (2024), and Ding et al. (2018) observed improved signal detectability at low doses (1 mg/kg), whereas Chu et al. (2016) reported cognitive improvements at higher doses. Additionally, Bhattacharya et al. (2015) found an age-related effect, with diminished drug efficacy in older subjects. In contrast, Benn and Robinson (2024) reported no significant effects of MPH on attentional performance. Overall, the administered dose and subjects’ baseline performance emerge as critical determinants of treatment outcomes, with a general pattern of low-dose enhancement and high-dose impairment or disruptive effects across most cognitive domains assessed.

## Discussion

This systematic review aimed to evaluate the impact of MPH on attentional processes in rats by analyzing the existing scientific literature. The findings obtained from the selected articles, following the established search protocol, reveal a heterogeneous landscape regarding the effects of MPH on attentional and related tasks. This variability in results can be attributed to differences in the dosages used, administration methods, and behavioral tests employed across studies (Appendix C).

Dosage considerations for MPH in rodents are crucial due to their higher metabolic rates. The literature commonly classifies dosages as low (below 5 mg/kg), comparable to human clinical cases, moderate (5 mg/kg to 10 mg/kg), and high (above 10 mg/kg) (Barron et al. 2009; Yang et al. 2010). In this review, dosages were adaptively classified based on the reported results: low (0.1 - 3 mg/kg), moderate (4 - 8 mg/kg), and high (9 - 30 mg/kg). Numerous studies have demonstrated that behavioral responses in animals vary significantly with dosage (Broussard et al. 2019; Venkataraman et al. 2019). The dosage ranges used in the selected articles align with those in other animal studies involving MPH (Barron et al. 2009; Berridge et al. 2006; Broussard et al. 2019; Devilbiss and Berridge 2008a; Gomes et al. 2010; Gray et al. 2007; Lages et al. 2021; LeBlanc-Duchin and Taukulis 2009; Levant et al. 2010; Medina et al. 2022; Panfil et al. 2023; Rostron et al. 2013; Salman et al. 2019, 2021; Sloan et al. 2016; Sontag et al. 2011; Thanos et al. 2010, 2015; Tian et al. 2009; Van Der Marel et al. 2014; Venkataraman et al. 2019; Yang et al. 2010).

The route of drug administration also influences its effects, as pharmacodynamic differences exist between intraperitoneal, subcutaneous, and oral administration. Panfil et al.(2023) reported that peak plasma concentrations of MPH are higher in animals receiving intraperitoneal injections. In this review, 10 studies administered MPH via injection: 8 used intraperitoneal administration (Andrzejewski et al. 2014; Ding et al. 2018; Galizio et al. 2014; Higgins et al. 2020; Motamedi et al. 2019; Ravichandran et al. 2015; Rowan et al. 2015; Tomlinson et al. 2014), and 2 used subcutaneous injections (Comeau and Kolb 2020; Takahashi et al. 2018).

This systematic review highlights the variability of MPH effects across different cognitive domains. While the primary emphasis was on sustained attention, the roles of related executive functions such as working memory and behavioral flexibility were considered due to their close interactions with attentional processes. Some studies reported memory improvements with dosages between 0.25 mg/kg and 2.5 mg/kg (Haleem et al. 2015; Zhou et al. 2017), while others found no significant effects with 3 mg/kg (Alfadly et al. 2014). These data align with Sloan et al. (2016), which observed that memory is enhanced by low doses of MPH and impaired by higher doses (2.5 mg/kg to 10 mg/kg). Only one study in this review reported improvements in memory and learning in healthy subjects with dosages from 2 mg/kg to 10 mg/kg, with 5 mg/kg being most effective; however, this dose also increased neuronal death (Ravichandran et al. 2015). Three articles found no influence of MPH on learning (Alfadly et al. 2014; Galizio et al. 2014; Motamedi et al. 2019), consistent with Rostron et al. (2013), who reported no learning improvement in healthy subjects.

Regarding working memory, the results were also diverse. Spencer and Berridge (2019) found improvements after intracerebral administration of 0.125 µg, while Comeau & Kolb (2020) reported impairment with 0.5 mg/kg. Sontag et al. (2011) observed working memory impairment with high doses but enhancement with low doses, as did Salman et al. (2021). Differences in administration age might explain these results, as Comeau and Kolb (2020) reported impairment in executive functions, particularly working memory, when MPH was administered from PND 21 to PND 100.

Subject age is a significant factor in result variability, as the maturation of prefrontal and fronto-striato-cerebellar circuits during development makes executive functions—including attention, working memory, and cognitive flexibility—particularly sensitive to MPH administration (Guo et al., 2023). Bhattacharya et al. (2015) found that MPH affects adult and aged rats differently. Studies by Levant et al. (2010) reported behavioral response differences in periadolescent and adult rats. Medina et al. (2022) observed age- and dose-dependent differences in behavioral response. Low doses (0.6 mg/kg) sensitized both adults and adolescents, while high doses (10 mg/kg) induced tolerance in adults. The 2.5 mg/kg group showed no significant differences in sensitization and tolerance between adolescents and adults, suggesting this medium dose may be optimal for both age groups. Similar tolerance and sensitization results were reported by Barron et al. (2009), who also noted that chronic MPH administration had different effects in adolescent and adult spontaneously hypertensive rats (SHR), indicating that treatment onset and exposure duration significantly influence response.

These differential results were explained by changes in pharmacokinetics and the development of dopaminergic systems in the Central Nervous System (CNS) (Gomes et al. 2010; Levant et al. 2010) or by structural and functional brain changes across the lifespan (Van Der Marel et al. 2014). Gray et al. (2007) provided anatomical evidence that chronic therapeutic MPH doses in juvenile rats result in short-term changes in four brain areas involved in motivated behaviors, cognition, appetite, and stress, but no significant long-term neuroanatomical changes.

This systematic review found that the vast majority of studies analyzed reported no improvements in spatial memory after MPH administration (Alfadly et al. 2014; Galizio et al. 2014; Motamedi et al. 2019), even though Tian et al. (2009) found improvement. This difference may be due to strain differences; Alfadly et al. (2014), Motamedi et al. (2019) used Wistar rats, Galizio et al. (2014) used Sprague–Dawley, and Tian et al. (2009) used SHR, an animal model of ADHD. Other studies reported spatial and recognition memory impairment with 5 and 10 mg/kg in non-SHR strains (LeBlanc-Duchin & Taukulis 2009). Sontag et al. (2011) also reported spatial memory impairment in Wistar rats, suggesting higher sensitivity to MPH. Thanos et al. (2010) found differences in MPH sensitivity between Wistar-Kyoto (WKY) and SHR rats, with WKY being more sensitive to 5 and 10 mg/kg doses, leading to sustained attention impairment. Yang et al. (2010) found that SD rats are most susceptible to MPH, even at low doses, while WKY rats are susceptible to high doses, and SHR rats are least susceptible.

Regarding locomotion, the reviewed articles reported dose-dependent increases (Beaudin et al. 2024; Bhattacharya et al. 2015; Haleem et al. 2015; Kim et al. 2016; Zhou et al. 2017). Beaudin et al. (2024) and Bhattacharya et al. (2015) found that the highest doses (3 mg/kg and 10 mg/kg, respectively) increased locomotion. Lages et al. (2021) found that doses above 3 mg/kg led to cognitive impairment and increased behavioral agitation. Thanos et al. (2015) reported increased hyperactivity with high doses, consistent with these authors. However, Haleem et al. (2015), Kim et al. (2016) and Zhou et al. (2017) reported reduced hyperactivity with 0.5 mg/kg and 1 mg/kg.

Most studies reported attentional improvements with MPH (Andrzejewski et al. 2014; Beaudin et al. 2024; Bhattacharya et al. 2015; Chu et al. 2016; Higgins et al. 2020; Kim et al. 2016; Spencer & Berridge 2019; Takahashi et al. 2018; Tomlinson et al. 2014; Zubedat et al. 2015), with low doses being the most effective, albeit with exceptions. Bhattacharya et al. (2015) found that 6 mg/kg and 8 mg/kg doses improved attention in adult rats, and Chu et al. (2016) reported improvements with 8 mg/kg in groups with low baseline performance. The results of Devilbiss & Berridge (2008b) and Lages et al. (2021) align with these findings, showing that low doses improve cognitive performance and working memory. Salman et al. (2019) also reported attentional improvements with low MPH doses. Berridge et al. (2006) found improvements in sustained attention with low doses.

In summary, both the existing literature and the articles in this systematic review show heterogeneous results after MPH administration. These differences may be due to rat strain, with SHR being less susceptible and Wistar/WKY more sensitive, especially at high doses. Low doses improve cognitive performance, memory, and attention, while doses above 3 mg/kg impair them. Low doses reduce locomotor hyperactivity, and high doses increase it. Studies with divergent results are often related to administration age, with juvenile rats being more susceptible and aged rats less benefited by MPH, due to the development of CNS neuronal circuits.

### Theoretical implications

The different effects of MPH on cognition and locomotion suggest a complex interaction of the drug with neuronal circuits. According to Spencer & Berridge (2019), the brain area of administration influences the effects, with the medial PFC being the area associated with attentional improvements. However, high doses of the drug are associated with an increase in locomotor activity. A detailed analysis of dopaminergic and noradrenergic circuits could improve the understanding of attention, motor skills, and executive functions. Likewise, it would be valuable to further investigate the PFC and its connection to various cognitive functions, as Devilbiss & Berridge (2008a) point out that the PFC exhibits a unique sensitivity to MPH.

Some of the studies reviewed in this research found no improvement or impairment in learning following MPH administration in healthy subjects (Alfadly et al. 2014; Galizio et al. 2014; Motamedi et al. 2019). The research on the effects of this psychostimulant in healthy individuals would be particularly relevant, given that neuroimaging evidence suggests alterations in neural activity in the PFC after MPH administration in both healthy subjects and those with ADHD (Berridge et al. 2006). This approach could provide deeper insight into the neural circuits involved in learning.

Differences between strains and doses may reflect variations in dopamine regulation or transmission. The differential effect according to age indicates critical periods in which MPH could damage cognition. This is relevant for clinical treatments, which often begin at early ages. Treatment individualization is necessary, as MPH acts differently according to the dose and function studied. Analyzing optimal doses according to patient deficits and age would be valuable.

Examining the effects of MPH in control animals provides critical insight into the drug's fundamental pharmacological actions on attentional systems. Control rodents offer a stable baseline for evaluating neurochemical and behavioral responses without the confounds introduced by pathology-related alterations (Berridge & Devilbiss 2011). Baseline-dependent effects are particularly informative because both animal and human research demonstrate that stimulant efficacy follows an inverted-U profile, with individuals or animals showing lower baseline performance exhibiting the greatest improvements (Cools & D'Esposito 2011). Moreover, the catecholaminergic and frontostriatal circuits modulated by MPH in rodents are evolutionarily conserved and operate similarly in both neurotypical populations and individuals with ADHD (Arnsten & Rubia 2012). Thus, findings in control animals help delineate core mechanisms whereby MPH enhances—or disrupts—attentional function, informing both preclinical interpretation and translational relevance.

### Strengths and limitations

One of the key strengths of this review is the comprehensive analysis of the scientific literature and the detailed classification of studies based on dosage, route of administration, rat strain, and age at administration. This classification has made it possible to identify general patterns regarding the effects of MPH on attention and other cognitive processes.

However, there are also notable limitations. One of the main challenges is the scarcity of studies that assess attention exclusively through specific behavioral tests. As a result, this review includes studies that analyze related functions, which could introduce some heterogeneity into the findings. Studies were included when their results were explicitly linked to attention, given that attentional capacity is closely associated with other cognitive abilities such as learning and working memory.

Another significant limitation is the lack of studies on female subjects. No articles evaluating the effects of MPH on attention in female rats were found between 2014 and 2024, making it difficult to generalize the findings to the female clinical population. One of the studies analyzed in this systematic review explicitly states that female subjects were not included due to the challenge of controlling variables such as the estrous cycle in attentional performance (Takahashi et al. 2018).

However, recent evidence suggests that the influence of the estrous cycle on behavior may be less significant than previously thought. Levy et al. (2023) reported that in mice, spontaneous behavior reflects individual variability rather than estrous state, and that females exhibit more stable and less variable exploratory behavior than males. It should be noted, however, that Levy et al. (2023) employed a mouse model rather than rats, did not use pharmacological interventions, and assessed only spontaneous behavior rather than attentional or cognitive performance under drug administration, limiting the direct extrapolation of their findings to rat models. Some research has begun to explore sex differences following MPH administration, such as Montiel-Herrera et al. (2024), which focuses on differences in prepulse inhibition after chronic and acute MPH administration, highlighting the importance of including female subjects and analyzing sex-specific responses in future studies.

An important limitation of studies using animal models of attention is methodological heterogeneity, including differences in behavioral tasks, MPH doses, age, and strain of the subjects, as well as small sample sizes that reduce statistical power and the generalizability of findings. These limitations persist due to the predominance of tasks such as the SAT and the 5-CSRTT, and the lack of standardized protocols for both dosing and task design, which hinders cross-study comparisons and their extrapolation to humans (Salmon et al. 2024). To ensure that animal models provide clinically useful information, it is essential that behavioral tasks are standardized, comparable across species, sensitive to experimental manipulations, and reproducible, allowing for the investigation of the neurobiological mechanisms of attention and the evaluation of translatable therapeutic strategies. Incorporating baseline-dependent analyses and advanced statistical adjustments improves the interpretation of drug effects on individual performance, maximizing information while minimizing regression-to-the-mean artifacts (Roberts & Young 2022).

In this context, future research should focus on how pharmacological modulation of attention can be integrated with therapeutic strategies aimed at enhancing higher-order cognitive functions and emotional regulation in patients. Attentional deficits observed in ADHD, anxiety, depression, or following brain injury directly impact working memory, processing speed, and executive planning, highlighting the importance of interventions that combine cognitive training with pharmacological support (Van der Velden et al. 2023). Another area of interest is the personalization of interventions, as the response to MPH and attentional therapies depends on individual factors such as age, treatment history, deficit severity, and patient motivation. Future studies could develop clinical criteria based on cognitive and neurophysiological profiles, enabling the adjustment of dosage, duration, and intensity of treatment, as well as the selection of the most effective training techniques for each individual, including cognitive rehabilitation strategies such as biofeedback, with baseline assessment to monitor individual improvements (Hopstaken et al. 2015).

### Clinical and translational implications for ADHD dosage and treatment

Animal models using sustained attention paradigms such as the 5C-CPT and rCPT, have been essential for understanding ADHD’s etiology, neurobiology, and pharmacological treatment, despite limitations including long training requirements and potential procedural biases (Roberts & Young 2022). The SHR, the most extensively studied model, exhibits reduced temporal sensitivity to reinforcers, dopaminergic hypofunction in prefrontal and striatal regions, and high intra-individual variability—traits supporting its relevance for investigating ADHD mechanisms and genetic contributions (Dela Peña et al. 2019; Denney 2001; Perry et al. 2010).

Alternative rat models have furthered understanding of ADHD subtypes and individual phenotypes. For instance, Sprague–Dawley rats exposed to neonatal hypoxia, modeling extreme prematurity, show dopaminergic neuron loss in the right ventral tegmental area and display hyperactivity and impulsivity as adults, resembling the hyperactive/impulsive subtype (Kohe et al. 2023). Similarly, genetically heterogeneous Sprague–Dawley rats with low baseline performance in attention and cognitive flexibility tasks exhibit phenotypes resembling human inattention and rigidity (Chu et al. 2016), and individual variation in conditioned responses—such as sign-tracking versus goal-tracking phenotypes—reflects distinct dopaminergic processing patterns that may underlie differential susceptibility to impulsivity (Serrano-Barroso et al. 2019). These findings support the notion that ADHD traits lie on a continuum and may result from either excessive or deficient dopaminergic transmission.

High-Active mice further illustrate the principle that ADHD involves catecholaminergic system dysregulation. Low-dose d-amphetamine selectively reduces impulsivity in these animals—not in controls—demonstrating an inverted-U dose–response curve and indicating that psychostimulants normalize hypoactive systems while potentially impairing performance at normal or elevated baseline function (Majdak et al. 2016). Pharmacologically, methylphenidate, amphetamine, and atomoxetine enhance dopaminergic and noradrenergic signaling across animal models, restoring behavioral and neurobiological deficits in proportion to baseline system dysfunction (Denney 2001; Dela Peña et al., 2019; Kohe et al. 2023). This baseline-dependent effect—central to the inverted-U hypothesis—suggests that stimulants function as neurochemical regulators rather than nonspecific activators.

Critically, developmental timing modulates treatment response. Because preclinical data from juvenile and adult animals may not fully extrapolate to pediatric human populations, potential long-term effects of early methylphenidate exposure warrant careful consideration. Emerging evidence indicates that sustained stimulant administration can produce persistent alterations in prefrontal synaptic plasticity, neuronal excitability, and working memory function (Urban et al. 2012). These findings collectively support a clinical approach emphasizing low initial doses with gradual, individually titrated escalation based on baseline cognitive and behavioral performance—a strategy designed to optimize attentional gains while minimizing over-stimulation and associated neurotoxic risks.

## Conclusions

In conclusion, the findings of this systematic review highlight the complexity of MPH’s effects on attention and other cognitive processes. The data suggest that low doses of MPH enhance attentional performance and working memory, whereas high doses may lead to attentional impairment and increased locomotor activity. Factors such as strain, age at administration, and route of administration play a crucial role in shaping the drug’s effects. Notably, Wistar and WKY strains appear to be the most susceptible to MPH’s influence. Future research should prioritize the inclusion of female subjects and the standardization of experimental protocols to improve the comparability of results.

## Supplementary Information

Below is the link to the electronic supplementary material.

Supplementary file1 (DOCX 23 KB)

Supplementary file2 (DOCX 22 KB)

Supplementary file3 (DOCX 51 KB)

Supplementary file4 (DOCX 19 KB)

## Data Availability

All data generated or analyzed during this systematic review are included in this manuscript and its supplementary materials. The original datasets supporting the conclusions of this manuscript were derived from publicly available sources cited in the reference list.
