# Supplementary material for: Methylphenidate and sustained attention in rats: a systematic review
Source: Psychopharmacology (Berl). 2026 Feb 13;243(9):2005–18. doi: 10.1007/s00213-026-07007-w (PMC13601167; doi:10.1007/s00213-026-07007-w)
Supplement: Supplementary file 1 [file 213_2026_7007_MOESM1_ESM.docx]

**Appendix A *-*** Risk of Bias tool (Rob) *Muftuoglu et al. (2014)*

|  | Alfadly et al. (2015) | Andrzejewski et al. (2014) | Beaudin et al. (2023) | Benn y Robinson (2024) | Bhattacharya et al. (2015) | Chu et al. (2016) | Comeau y Kolb (2020) | Ding et al. (2018) | Galizio et al. (2014) | Haleem et al. (2015) | Higgins et al. (2020) | Kim et al. (2016) | Motamedi et al. (2019) | Ravichandran et al. (2015) | Rowan et al. (2015) | Spencer y Berridge (2019) | Takahashi et al. (2018) | Tomlinson et al. (2014) | Zhou et al. (2017) | Zubedat et al. (2015) |
| --- | --- | --- | --- | --- | --- | --- | --- | --- | --- | --- | --- | --- | --- | --- | --- | --- | --- | --- | --- | --- |
| 1 |  |  |  |  |  |  |  |  |  |  |  |  |  |  |  |  |  |  |  |  |
| 2 |  |  |  |  |  |  |  |  |  |  |  |  |  |  |  |  |  |  |  |  |
| 3 |  |  |  |  |  |  |  |  |  |  |  |  |  |  |  |  |  |  |  |  |
| 4 |  |  |  |  |  |  |  |  |  |  |  |  |  |  |  |  |  |  |  |  |
| 5 |  |  |  |  |  |  |  |  |  |  |  |  |  |  |  |  |  |  |  |  |
| 6 |  |  |  |  |  |  |  |  |  |  |  |  |  |  |  |  |  |  |  |  |
| 7 |  |  |  |  |  |  |  |  |  |  |  |  |  |  |  |  |  |  |  |  |
| 8 |  |  |  |  |  |  |  |  |  |  |  |  |  |  |  |  |  |  |  |  |
| 9 |  |  |  |  |  |  |  |  |  |  |  |  |  |  |  |  |  |  |  |  |
| 10 |  |  |  |  |  |  |  |  |  |  |  |  |  |  |  |  |  |  |  |  |

*The boxes are answered with colors. Green: YES, Red: NO, and Orange: Unclear/Doubtful.*

*.*

**Selection bias**
*Sequence generation*

1.Describe the methods used, if any, to generate the allocation sequence with enough detail to allow an assessment of whether comparable groups should be produced. - Was the allocation sequence properly generated and applied?

*Baseline characteristics*

2. Describe all potential prognostic factors or characteristics of the animals, if any, that are compared to assess whether the intervention and control groups were similar at the start of the experiment. - Were the groups similar at baseline or adjusted for confounding factors in the analysis?
*Allocation concealment*

3.Describe the method used to conceal the allocation sequence with enough detail to determine if the intervention assignments could have been predicted before or during enrollment. - Was allocation adequately concealed?

**Performance bias**
*Random housing*
4. Describe any measures used, if any, to house animals randomly within the animal room. - Were the animals randomly housed during the experiment?
*Blinding*
5. Describe any measures used, if any, to blind caregivers and researchers of the trial so they did not know which intervention each animal received. Provide any information related to the effectiveness of the anticipated blinding.- Were caregivers and/or researchers blinded to which intervention each animal received during the experiment?

**Detection bias**
*Random outcome assessment*
6. Describe if animals were randomly selected for outcome assessment, and what methods were used to select the animals, if any. - Were animals randomly selected for outcome assessment?
*Blinding*
7. Describe any measures used, if any, to blind outcome assessors so they did not know which intervention each animal received. Provide any information related to the effectiveness of the anticipated blinding. - Was the outcome assessor blinded?

**Attrition bias**
*Incomplete outcome data*
8. Describe the completeness of the outcome data for each primary outcome, including loss to follow-up and exclusions from the analysis. Indicate whether loss to follow-up and exclusions were reported, the numbers in each intervention group (compared with the total number of animals randomized), reasons for loss to follow-up or exclusions, and any re-inclusions in the analyses for review. - Were incomplete outcome data adequately addressed?

*Reporting bias*
Selective outcome reporting
9. Indicate how selective outcome reporting was examined and what was found. - Are the study reports free from selective outcome reporting?

**Other**
10. Indicate any significant concerns about biases not covered by other domains in the tool. - Did the study appear to be free from other issues that could result in a high risk of bias?
