# Supplementary material for: Methylphenidate and sustained attention in rats: a systematic review
Source: Psychopharmacology (Berl). 2026 Feb 13;243(9):2005–18. doi: 10.1007/s00213-026-07007-w (PMC13601167; doi:10.1007/s00213-026-07007-w)
Supplement: Supplementary file 2 [file 213_2026_7007_MOESM2_ESM.docx]

**Appendix B:** CAMARADE checklist

|  | Alfadly et al. (2015) | Andrzejewski et al. (2014) | Beaudin et al. (2023) | Benn y Robinson (2024) | Bhattacharya et al. (2015) | Chu et al. (2016) | Comeau y Kolb (2020) | Ding et al. (2018) | Galizio et al. (2014) | Haleem et al. (2015) | Higgins et al. (2020) | Kim et al. (2016) | Motamedi et al. (2019) | Ravichandran et al. (2015) | Rowan et al. (2015) | Spencer y Berridge (2019) | Takahashi et al. (2018) | Tomlinson et al. (2014) | Zhou et al. (2017) | Zubedat et al. (2015) |
| --- | --- | --- | --- | --- | --- | --- | --- | --- | --- | --- | --- | --- | --- | --- | --- | --- | --- | --- | --- | --- |
| **1** | X | X | X | X | X | X | X | X | X | X | X | X | X | X | X | X | X | X | X | X |
| **2** | X | X | X | X | X | X |  | X | X | X | X | X | X | X |  |  |  | X | X | X |
| **3** | X | X | X | X |  |  | X | X | X | X | X | X | X | X | X | X | X | X | X | X |
| **4** |  |  |  |  |  |  |  |  |  |  |  |  |  |  |  |  |  |  |  |  |
| **5** |  |  |  |  |  |  |  |  |  |  |  |  |  |  |  |  |  |  |  |  |
| **6** | N/A | N/A | N/A | N/A | N/A | N/A | N/A | N/A | N/A | N/A | N/A |  | N/A | N/A | N/A | X | X | N/A | N/A | N/A |
| **7** |  |  |  | X |  | X |  | X |  |  |  | X |  |  |  |  | X | X | X |  |
| **8** |  |  |  | X | X |  |  |  |  |  | X |  |  |  |  |  |  |  |  |  |
| **9** | X | X | X | X | X | X | X | X |  | X | X | X | X | X | X | X |  | X | X | X |
| **10** |  |  | X | X |  |  |  |  |  | X | X |  | X | X | X | X | X | X |  | X |
| **Total** | 4 | 4 | 5 | 7 | 4 | 4 | 3 | 5 | 3 | 5 | 6 | 5 | 5 | 5 | 4 | 5 | 5 | 6 | 5 | 5 |

*It is answered with an "X" in the table if the answer is YES. If the question does not apply, we write "N/A", which means "Not Applicable".*

*In the total, the number of "X"s that the study meets is indicated.*

1. **Peer-reviewed publication:** Has the study been published in a peer-reviewed journal?
2. **Temperature control:** Is temperature control mentioned during the experiments?
3. **Random treatment or control assignment:** Is a process of random assignment or control described?
4. **Allocation concealment:** Was the treatment administered in a blinded manner?
5. **Blinded outcome assessment:** Were the outcomes assessed in a blinded manner?
6. **Use of anesthetic with no significant intrinsic neuroprotective activity:** Was any anesthetic used that has no significant intrinsic neuroprotective activity?
7. **Animal model (aged, diabetic, or hypertensive):** Were specific animal models (aged, diabetic, or hypertensive) used?
8. **Sample size calculation:** Was a sample size calculation performed before starting the study?
9. **Animal welfare regulations:** Were animal welfare regulations followed during the conduct of the study?
10. **Conflict of interest:** Was a statement regarding potential conflicts of interest included in the study?
