# Supplementary material for: Methylphenidate and sustained attention in rats: a systematic review
Source: Psychopharmacology (Berl). 2026 Feb 13;243(9):2005–18. doi: 10.1007/s00213-026-07007-w (PMC13601167; doi:10.1007/s00213-026-07007-w)
Supplement: Supplementary file 3 [file 213_2026_7007_MOESM3_ESM.docx]

**Appendix C -** Results table

| **Study** | **Objective** | **Subjects** | **Drug** | **Procedure** | **Results** | **Discussion** |
| --- | --- | --- | --- | --- | --- | --- |
| 1. Alfadly et al. (2015) | Compare the effect of Catha edulis and methylphenidate on memory, learning and attention. | Wistar male rats (6-8 weeks) | C.edulis and MPH. Oral gavage, 30 min before.  MPH dose: 3 mg/kg. | Radial Arm Maze (RAM) | *RAM*. MPH outperformed control in latency. No effect on reference memory. | MPH increase alertness and wakefulness, reducing input latency. Improves attention although is does not affect memory or learning. |
| 2. Andrzejewski et al. (2014) | Analyse the effects of low-dose psychostimulants on attention and their relation to the treatment of ADHD. | Sprague-Dawley male rats (9 months). | AMPH and MPH Intraperitoneal (IP) injections, 30 min before.  MPH dose: 0.5 mg/kg and 1 mg/kg. | Sustained Attention (SA): Signal detection procedure.  Low rate differential reinforcement schedule (DRL). | In SA, MPH enhanced detectability without altering response bias, with 0.5 mg/kg MPH and control showing similar effects. In DRL, response rates stabilized after 32 sessions, with no significant drug effects. | Low doses of MPH improve signal detection, not inhibition. In SA, improved attention in unaffected rats. |
| 3. Beaudin et al. (2023) | Investigate MPH effects on rats exposed to Mn during development, which causes attentional and sensorimotor deficits similar to ADHD. | Long-Evans male and female rats | Oral administration with biscuits. 30 min (attention)/ 90 min before (sensorimotor).  MPH dose: 0.5mg/kg, 1.5 mg/kg or 3.0mg/kg. | 5CSRTT  The Montoya staircase test | MPH ameliorates Mn-induced attentional dysfunction at low doses (0.5mg/kg). Doses of 3mg/kg increase impulsivity.  High doses of MPH improved sensorimotor deficits caused by Mn | MPH attenuates Mn effects in a dose- and region-dependent manner, enhancing attention at low doses and acting early on the sensorimotor area at high doses. |
| 4. Benn y Robinson (2024) | Evaluate the effects of different drugs on a tactile task based on RSVP. | Lister hooded male rats | Oral MPH (1 to 10 mg/kg) with strawberry milkshake, 30 min before.  Also: amphetamine, atomoxetine, ketamine, nicotine. | Rapid Serial Visual Presentation in (R-RSVP). | All groups performed similarly, and MPH had no significant effect. | The analysis showed a reduction in accuracy over time. MPH had no significant effect. |
| 5. Bhattacharya et al. (2015) | Investigate the effects of MPH on Sustained Attention in adult and elderly rats. | Sprague-Dawley male rats. Adult and advanced age | MPH orally with cereal, 15 min prior to testing.  Dose range 2.0mg/kg-12.0mg/kg. | Sustained Attention Task + Visual Distraction  Open Field | MPH (6 and 8 mg/kg) improved performance in adults but had no effect in the elderly, who also showed high variability. Visual distraction reduced LV scores in both groups; 8 mg/kg MPH reversed this deficit in adults but not in the elderly. Locomotor activity increased at 10 mg/kg in adults, with no change in the elderly. | Elderly rats perform comparably to adult rats; however, the effect of MPH on vigilance diminishes with age. While the distractor impairs performance in both groups, MPH only restores function in adults. Therefore, it may not be effective as a cognitive enhancer in the elderly. |
| 6. Chu et al. (2016) | Understanding the cognitive improvements of MPH on sustained attention in rats with different levels of attention. | Sprague-Dawley male rats | MPH or oral saline via sweetened cereal, 15 min before.  Doses of MPH: 2, 8 and 16 mg/kg | Sustained Attention Task  Attention set shifting apparatus | In SA, 8 mg/kg MPH improved performance in low performers, with no effect on high performers. In Attention Set-Shifting, MPH enhanced performance in low performers but impaired flexibility in high performers. | The effect of MPH on sustained attention varied according to performance and was maintained in the attentional switching task. The optimal dose in low performance was 8 mg/kg. |
| 7. Comeau y Kolb (2020) | Investigate persistent changes in executive functions and neuronal morphology due to juvenile MPH consumption. | Long-Evans male rats.  Age: 21 PND – 100 PND | Subcutaneous injections, twice daily for 37 consecutive days.  MPH:(0.5mg/kg)  d-amphetamine (1.0 mg/kg) | Play behavior  T-Maze  Open field (OF) | In the OF, MPH had no immediate effect, but increased activity after six days. MPH rats made twice as many errors in the T-maze and initiated fewer play interactions than controls. | Early exposure to MPH impacts behavior and neural circuitry in the CPF. In working memory tasks, treated rats exhibited more errors than controls. |
| 8. Ding et al. (2018) | Investigate the effect of pro-attenuating drugs on CPT in rodents. | Listere-Hooded male rats | Intraperitoneal injections, 30 min before. MPH, ABT-594, modafinil, atomoxetine, donepezil and memantine.  MPH doses: 0.3, 1 and 3 mg/kg. | CPT | MPH doses of 1 and 3 mg/kg increased hits. All doses raised false alarms and lowered the C-score. Discrimination worsened at 3 mg/kg MPH, which also increased blank touches and reduced correct response latency. | MPH at high doses increased hits, false alarms, and blank touches, decreased correct latency, and affected discriminability (d'), suggesting disinhibition and impulsivity. |
| 9. Galizio et al. (2014) | Investigate the effects of MDMA, MA and MPH in a RAP adaptation of the Morris Swimming Task (MST). | Holtzman Sprague-Dawley male rats. Age 90-120 PND. | Intraperitoneal injection, 15 min before. Twice a week.  MDMA, MA & MPH.  Dose MPH: 1.0 - 30.0 mg/kg. | MST | Only the 17 mg/kg MPH dose differed significantly from saline, and 30 mg/kg MPH affected latencies. | None of the drugs produced selective effects for place learning; high doses caused difficulties in swimming. |
| 10. Haleem et al. (2015) | Examine the effects of clinically relevant doses of MPH over a prolonged period on cognition and emotions. | Wistar male rats | Oral MPH, twice daily.  MPH doses: 0, 0.25, 0.5 and 1.0 mg/kg. | Open field  Activity Cage   Water maze | MPH increased motor activity at 0.5 mg/kg (cage) and 0.5-1 mg/kg (open field). In the Water Maze, it enhanced learning acquisition (0.25-0.5 mg/kg) and retention across all doses. Behavioral sensitization was observed long-term at 1 mg/kg. | All three doses equally impacted memory retention, extinction, and reconsolidation, while inhibiting habituation in the open field in a dose-dependent manner. The 1 mg/kg dose induced behavioral sensitization. Clinical doses of MPH enhance long-term memory. |
| 11. Higgins et al. (2020) | Determine the effect of AMP, MPH, NIC and ATX on attention, impulsive action and motivation. | Long Evans male rats. Age (4–14 month) | Intraperitoneal injections, 30 min before.  d-Amphetamine sulphate (AMP), Methylphenidate hydrochloride (MPH), Atomoxetine hydrochloride (ATX).    Dose range MPH: 1-6 mg/kg | 5-CSRTT  Go/NoGo Task  Progressive Reinforcement Programme  Locomotor activity | 5-CSRTT: MPH improved dose-dependent performance, increasing accuracy and speed while reducing missed trials. It heightened premature responses, especially at low performance levels, and did not improve overall accuracy but reduced premature responses with short stimuli. Decreased premature responses at high impulsivity and increased them at low impulsivity.  Go/NoGo: MPH improved Go trial performance but had no effect on accuracy, false alarms, or latencies. There was a tendency to increase lever presses at 6-10 mg/kg. | 5-CSRTT: Increased complexity reduced accuracy and heightened premature responses, impacting attention and response control.  Drugs: MPH improved accuracy with short TIs; low doses may enhance attentional performance. |
| 12. Kim et al. (2016) | Preclinical rationale for rTMS in ADHD and neurochemical mechanisms of MPH and rTMS. | SHR male rats Wistar-Kyoto male rats Juvenile stage | Oral administration, twice daily.  MPH: 1mg/kg | Open field Y-maze Elevated plus maze | *Open field:* MPH travels less distance than sham grou*p*.  *Y- maze:* No difference in the second week; in the third week, MPH improves spontaneous alternation. | MPH improves spontaneous alternation, suggesting improved attention. It also reduces hyperactivity according to distance travelled but increases anxiety. |
| 13. Motamedi et al. (2019) | Evaluate the co-administration of marijuana and MPH on learning and spatial memory. | Wistar male rats. Adults | Intraperitoneal injection, 30 min before.  Marihuana and MPH.  MPH Doses: 0.5, 1.0, 2.5, 5.0 and 10 mg/kg. | Water Maze (WM) | *WM*: MPH does not affect learning and memory. | MPH did not affect spatial learning, but 10 mg/kg increased swimming speed on day 2. |
| 14. Ravichandran et al. (2015) | Evaluate MPH treatment effects on hippocampal and dentate gyrus neuronal morphology and cognition | Wistar male rats. Age: 2 months | Intraperitoneal injections, twice daily for 10 days.  MPH dose: 2 mg/kg and 5 mg/kg | Condition avoidance test Histomorphological study | Both doses improve learning compared to control, with the 5 mg/kg dose being more effective. Both doses also significantly enhance retention and reassessment. | MPH improves learning and retention in normal rats.  The 5 mg/kg dose significantly reduced the number of neurons. |
| 15. Rowan et al. (2015) | Examine the effects of adolescent MPH exposure on adult cognition. | Long Evans male rats. Age: 25 PND | Intraperitoneal injections, 5 days a week for 5 weeks  MPH dose 20.0mg/kg | Task EMS | MPH influenced the learning of boundary elements and violated items, without affecting within-segment performance. It impaired boundary elements on certain days. | Chronic exposure to high doses of MPH in adolescence causes persistent effects on learning, affecting Stimulus-Response and multiple cues, but not abstract rules. |
| 16. Spencer y Berridge (2019) | Investigate the neural mechanisms of the cognitive effects of different doses of psychostimulants. | Sprague-Dawley male rats (male) | Via bilateral cannulae in the CPFm. Atipamezole, Benoxathian, Muscimol and MPH.  MPH HCl doses: 0.03 µg, 0.125 µg, 0.5 µg, 2.0 µg/500 nl. | Surgery Sustained Attention task (SAT) T-maze | MPH: In CPFdm, dose–response reversal improved, peaking at 0.5 µg/hemisphere. Working memory and SAT also improved. Optimal doses: 0.5 µg/g for attention, 0.125 µg/g for memory. | SAT and working memory rely on frontostriatal regions. MPH improves SAT specifically in the CPFdm, requiring four times higher doses than for working memory. MPH effects involve α2 and D1 receptors in the CPF, while α1 receptors selectively enhance SAT. |
| 17. Takahashi et al. (2018) | Investigate the neurochemical and neurophysiological profiles of ASP 2905 and its effects on attention. | ddY mice; SHRSP and Wistar-Kyoto rats (4 weeks old); Wistar/ST and SD rats (8-9 weeks old). | MPH subcutaneously SD rats (1 mL/kg) - SHRSP and WKY rats (2 mL/kg)  Other drugs: Atomoxetine,  Amphetamine, ASP 2905 and guanfacine | Five-trial passive avoidance task (5T-PAT)  Microdialysis  Electroencephalogram | Shorter cumulative latencies in SHRSP than in WKY.  MPH significantly increased latencies in juvenile SHRSP versus controls. EEG showed increased alpha power with methylphenidate. | MPH increased cumulative latency and alpha power—associated with alertness in humans—in SHRSP rats, while also improving attention and reducing impulsivity. |
| 18. Tomlinson et al. (2014) | Assess the consequences of pharmacotherapies in subgroups. | Adult female Lister-hooded rats classified into low (LA) and high attention (HA) and low (LI) and high impulsivity (HI) | Intraperitoneal injections, 30 min before.  Atomoxetine and MPH.  MPH doses: 0.5mg/kg, 1 mg/kg and 2mg/kg. | 5-CSRTT | MPH improved accuracy in LA animals, reaching HA performance levels at 2 mg/kg.  HI animals showed more premature responses than LI. MPH (0.5 and 1 mg/kg) significantly reduced premature responses in HI, while 2 mg/kg increased them in LI. | The MPH has a differential impact depending on the animal's baseline performance, as well as attentional and impulsive symptomatology. |
| 19. Zhou et al. (2017) | Analyse the effect of baicalin on hyperactivity, impulsivity and inattention. | SHR male rats , 4 weeks old  Wistar Kyoto male rats (WKY) | Administration by gavage, 2 doses daily for 4 weeks.  Baicalin and MPH  MPH dose: 0.007mg/mgl. | Open field  Morris Maze | MPH significantly increased distance, with a reduction in from Days 14-28. MPH decreased hyperactivity, increased attention, and reduced distances and rings.  Morris maze: MPH significantly reduced latency. | MPH regulate movement, learning and memory in SHR rats, reducing hyperactive, inattentive and impulsive symptoms of ADHD. |
| 20. Zubedat et al. (2015) | Examine the effects of environmental enrichment (EE) and prenatal stress (PS) on types of care, and the possible mediating/moderating effect of MPH. | Wistar male rats. Age: 60 - 90 PND | Oral administration by pipette, 30 min before.  Dose MPH:2.7mg/kg. | Prenatal Stress: swimming stress, mirror stress and restraint stress  Environmental enrichment  Open field test  Object recognition  Rotor-rod  Prepulse inhibition | *Object recognition*: EE x MPH interaction shows greater exploration than control.  *Novel object recognition*: MPH, EE and EE+MPH improve vs. PS.  *Pre-pulse inhibition (PPI)*: PS performs worst, EE best. MPH improves at intermediate intensities.  MPH and/or EE increase IPP vs. control. | PS) partially impairs attention, but both EE and MPH mitigate these effects, showing a positive interaction. EE improves IPP and object recognition, while MPH enhances IPP and selective attention. Both increase neurotrophic factors, indicating a shared mechanism against PS, suggesting EE and MPH as potential therapeutic interventions. |
