# Supplementary material for: Methylphenidate and sustained attention in rats: a systematic review
Source: Psychopharmacology (Berl). 2026 Feb 13;243(9):2005–18. doi: 10.1007/s00213-026-07007-w (PMC13601167; doi:10.1007/s00213-026-07007-w)
Supplement: Supplementary file 4 [file 213_2026_7007_MOESM4_ESM.docx]

**Table 2**

*Summary of animal studies using behavioral attention tasks with MPH*

| **Author & Year** | **Sample Size** | **Age** | **MPH Dose** | **Behavioral attention Test** | **Statistical Significance*** | **Risk of Bias**** |
| --- | --- | --- | --- | --- | --- | --- |
| Andrzejewski et al. (2014) | n=8  Male Sprague-Dawley rats | 9 months | MPH: 0.5 & 1.0 mg/kg  IP (intraperitoneal) | Sustained Attention (SA): Signal detection procedure. | d’ ↑, p < 0.05  False Alarms ↓, p < 0.05  0.5 mg/kg (F1,7=15.92)  1.0 mg/kg (F1,7=12.23) | High |
| Beaudin et al. (2023) | N=128  Male Long-Evans | PND 109 | MPH: 0.5, 1.5, o 3.0 mg/kg  Oral via cereal | 5-Choice Serial Reaction Time Task, 5-CSRTT | Mn-exposed x 0.5 mg/kg % Accuracy, *p* < 0.05, (F(15,747) = 4.82, *p* < 0.0001) | Low |
| Bhattacharya et al. (2015) | N=10  Male  Sprague-Dawley | 6-12 months  18-22 months | MPH: 2.0 - 12.0 mg/kg  Oral via cereal | Sustained Attention Task (SAT) | Adults rats:  MPH 6.0 & 8.0 mg/kg (oral)↑ Vigilance Index (VI), p < 0.05  MPH 8.0 mg/kg (oral) ↑ VI, t = 3.42, p < 0.01 | High |
| Chu et al. (2016) | N=25  Male Sprague–Dawley | Age not specified | MPH: 2, 8 & 16 mg/kg  Oral via cereal | Sustained Attention Task | 8 mg/kg MPH ↑ significant, p < 0.01 (F(1,24) =7.995) | Moderate |
| Ding et al. (2018) | N=8  Male  Lister-Hooded | Age not specified | MPH0.3, 1, & 3 mg/kg  IP | Rodent touchscreen-based Continuous Performance Test, CPT | MPH treatment ↓ significant d’ (F (3,141) =4.135, p<0.01) | High |
| Higgins et al. (2020) | N=40  Male  Long Evans | Varied from 4 to 14 months | MPH: 1–6 mg/kg  IP | 5-Choice Serial Reaction Time Task, 5-CSRTT | MPH ↑ % Hits, % Correct, Correct Latency, Omissions (P<0.001) | Low |
| Spencer y Berridge (2019) | N=21  Male Sprague–Dawley | Adults | 0.03, 0.125, 0.5, 2.0 µg/500 nL per hemisphere | Sustained Attention | MPH (dmPFC) inverted U improvement in d’, p < 0.01 (F4,66.8= 4.15, p < 0.01) | Moderate |
| Takahashi et al. (2018) | N=10 Male Stroke-Prone Spontaneousl & Hypertensive (SHRSP) y Wistar-Kyoto (WKY) | 4 weeks | MPH: 0.03, 0.1 & 0.3 mg/kg  Subcutaneous injection (SC) | Five-trial passive avoidance task (5T-PAT) | 0.1 & 0.3 mg/kg MPH ↑ cumulative latencies, SHRSP rats (0.1 mg/kg: p = 0.0097; 0.3 mg/kg: p = 0.0051) | High |
| Tomlinson et al. (2014) | N=40  Female Lister-hooded | Adults | MPH: 0.5, 1.0 & 2.0 mg/kg  IP | 5-choice Continuous Performance Test, 5C-CPT | 2.0 mg/kg MPH ↑ accuracy in LA rats vs. vehicle (p < 0.01)  0.5 & 1.0 mg/kg MPH ↑ Vigilance Index in LA rats (p < 0.01) | Moderate |

*Note. *This column reports the statistical significance of the behavioral tests used to assess attention, including only the conditions in which MPH produced significant improvements or impairments. **Risk classification based on sample size followed the Resource Equation, which suggests that (total experimental units – number of treatment groups) should fall between 10 and 20. Studies with N ≤ 10 were classified as high risk due to low statistical power; those with N = 11–39 as moderate risk; and those with N > 40 as low risk (Andrews et al., 2016; Consideration of Sample Size in Neuroscience Studies, 2020; Festing, 2018; de Vries et al., 2014). These thresholds were adapted to the context of animal research and aligned with the principles of the 3Rs (replacement, reduction, refinement), whose implementation also guides sample size considerations in systematic reviews (de Vries et al., 2014).*
